# Phonons in Copper Diphosphide (CuP_2_): Raman Spectroscopy and Lattice Dynamics Calculations

**DOI:** 10.1021/acs.jpcc.3c02108

**Published:** 2023-05-26

**Authors:** Mirjana Dimitrievska, Alexander P. Litvinchuk, Andriy Zakutayev, Andrea Crovetto

**Affiliations:** † Transport at Nanoscale Interfaces Laboratory, Swiss Federal Laboratories for Material Science and Technology (EMPA), Ueberlandstrasse 129, 8600 Duebendorf, Switzerland; ‡ Texas Center for Superconductivity and Department of Physics, University of Houston, Houston, Texas 77204-5002, United States; § Materials Science Center, National Renewable Energy Laboratory, Golden, Colorado 80401, United States; ∥ Centre for Nano Fabrication and Characterization (DTU Nanolab), Technical University of Denmark, 2800 Kongens Lyngby, Denmark

## Abstract

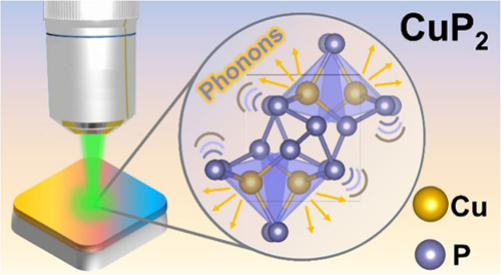

Copper diphosphide (CuP_2_) is an emerging binary semiconductor with promising properties for energy conversion and storage applications. While functionality and possible applications of CuP_2_ have been studied, there is a curious gap in the investigation of its vibrational properties. In this work, we provide a reference Raman spectrum of CuP_2_, with a complete analysis of all Raman active modes from both experimental and theoretical perspectives. Raman measurements have been performed on polycrystalline CuP_2_ thin films with close to stoichiometric composition. Detailed deconvolution of the Raman spectrum with Lorentzian curves has allowed identification of all theoretically predicted Raman active modes (9A_g_ and 9B_g_), including their positions and symmetry assignment. Furthermore, calculations of the phonon density of states (PDOS), as well as the phonon dispersions, provide a microscopic understanding of the experimentally observed phonon lines, in addition to the assignment to the specific lattice eigenmodes. We further provide the theoretically predicted positions of the infrared (IR) active modes, along with the simulated IR spectrum from density functional theory (DFT). Overall good agreement is found between the experimental and DFT-calculated Raman spectra of CuP_2_, providing a reference platform for future investigations on this material.

## Introduction

Copper diphosphide (CuP_2_) is an emerging binary semiconductor with versatile properties for various energy conversion and storage applications. It has shown promise as a component in composite anode materials for lithium- and sodium-based batteries due to its cyclability, capacity, and resistance to degradation.^1−5^ Its high optical absorption coefficient (above 10^5^ cm^–1^ in the visible region) and band gap of 1.5 eV make it a valuable candidate as an absorber in solar cells.^6,7^ Additionally, it has been investigated as a thermoelectric material^8,9^ and an electrocatalyst for hydrogen and oxygen evolution.^10^

While functionality and possible applications of CuP_2_ have been studied, there is a curious gap in the investigation of its fundamental properties. Specifically, insights into vibrational properties and phonon behavior of CuP_2_ are very few. Qi et al. have looked into the lattice anharmonicity of CuP_2_ by using first principle density functional theory (DFT) calculations and neutron scattering vibrational spectroscopy.^11^ They have reported temperature-dependent phonon density of states (PDOS) of CuP_2_ and revealed a rattling mode at around 90 cm^–1^ (11 meV), related to vibrations of Cu atomic dimers, as responsible for low lattice thermal conductivity. Recently, Crovetto et al.^6^ have reported the Raman spectrum of CuP_2_ along with a brief, qualitative discussion on the Raman peak positions. However, to date, there are no in-depth studies on Raman spectral behavior of CuP_2_. Raman spectroscopy is a powerful technique used for structural characterization, such as phase identification,^12−15^ crystal quality,^16^ and defect determination at the microscale,^17−21^ with shorter acquisition times when compared to other techniques. However, in order to be able to use Raman spectroscopy as a suitable tool for the above mentioned purposes, it is necessary to have reliable reference Raman spectra of the material, with detailed identification of all peaks and their vibrational origin.

In this work, we provide a reference Raman spectrum of CuP_2_, with a complete analysis of all Raman active modes from both experimental and theoretical perspectives. To the best of our knowledge, this is the first comprehensive analysis of the lattice vibrations in CuP_2_, along with a concise comparison of theoretical and experimental results in terms of phonon symmetries and frequencies. The experiments have been performed on polycrystalline CuP_2_ thin films with close to stoichiometric composition. Together with the detailed deconvolution of the Raman spectrum with Lorentzian curves, this has allowed identification of all theoretically expected Raman modes. Furthermore, calculations of the phonon density of states (PDOS), as well as the phonon dispersions, provide a microscopic understanding of the experimentally observed phonon lines, in addition to the assignment to the specific lattice eigenmodes. We further provide the theoretically predicted positions of the infrared (IR) active modes, along with the simulated IR spectrum. These results can be used as a reference for identification of the CuP_2_ phase, as well as for building methodologies for effective defect screening of bulk materials and films that might contain structural inhomogeneities.

## Materials and Methods

### Material Preparation

This study uses samples from ref (6), where the synthesis procedure developed by Crovetto et al. was described. Amorphous CuP_2+*x*_ thin films were deposited using reactive radio frequency (RF) sputtering on borosilicate glass over a 10 × 5 cm^2^ area. A Cu target and a Cu_3_P target were cosputtered at 2 Pa total pressure in a 5% PH_3_/Ar atmosphere without intentional heating and without substrate rotation. The targets were oriented so that one short side of the substrate would mainly be coated by the Cu target and the other short side by the Cu_3_P target. Immediately after deposition, CuP_2+*x*_ films were cut into smaller pieces and annealed in a lamp-based rapid thermal annealing (RTA) furnace in an N_2_ atmosphere. Elemental composition was determined by X-ray fluorescence (XRF) calibrated by Rutherford backscattering spectrometry (RBS). Samples with close to stoichiometric composition of [P]/[Cu] = 2 were used for Raman measurements.

### Characterization

X-ray diffraction (XRD) measurements were conducted with a Bruker D8 Discover diffractometer by using Cu Kα radiation, a fixed incidence angle of 10°, and a two-dimensional (2D) detector integrating the diffraction signal over a 72° *X* range for each value of 2θ. Structural analysis and phase identification were performed using the Le Bail refinement (profile matching method) in the FullProf software.^22^ In this analysis procedure, the structure factors *F*_*hkl*_, which are deduced from the given space group, are initially set to arbitrary values.^23^ They evolve iteratively according to the estimations obtained by apportioning data values among the contributing reflections. This results in the determination of phases present in the material and their unit cell parameters. Raman spectra were measured with a Renishaw inVia Raman microscope with 532 nm excitation wavelength and 4 W/mm^2^ power density at 50× magnification. Laser power conditions were selected based on a power study, which involved measuring Raman spectrum in the same point on the material with increasing laser power densities, starting from the lowest power available. For each laser power, the spectrum was monitored for changes in peak positions, peak widths, or appearance of new peaks. The highest power for which no changes in these parameters were observed was taken as the optimal laser power for measurements. Scanning electron microscopy (SEM) images were taken by a Hitachi S-3400N microscope at 5 kV beam voltage, using a field emission gun and a secondary electron detector. All measurements were performed within 24 h after annealing to avoid sample degradation.

### Lattice Dynamics Calculations

The first-principles calculations of the electronic ground state of CuP_2_ were performed within the local density approximation using Ceperley–Adler functional,^24,25^ as implemented in the CASTEP code.^26^ Norm-conserving pseudopotentials were used. The cutoff energy for the plane wave basis was set to 600 eV. A self-consistent field (SCF) tolerance better than 10^–7^ eV per atom and the phonon SCF threshold of 10^–12^ eV per atom were imposed. Prior to performing calculations, the structure was relaxed so that forces on atoms in the equilibrium position did not exceed 2 meV Å^–1^, and the residual stress was below 5 MPa. Experimentally determined lattice parameters were used as a starting point. An integration over the Brillouin zone was performed over a 3 × 3 × 2 Monkhorst-Pack grid in reciprocal space.

## Results and Discussion

### Structural and Morphological Assessment of CuP_2_ Thin Film

**Figure 1:**
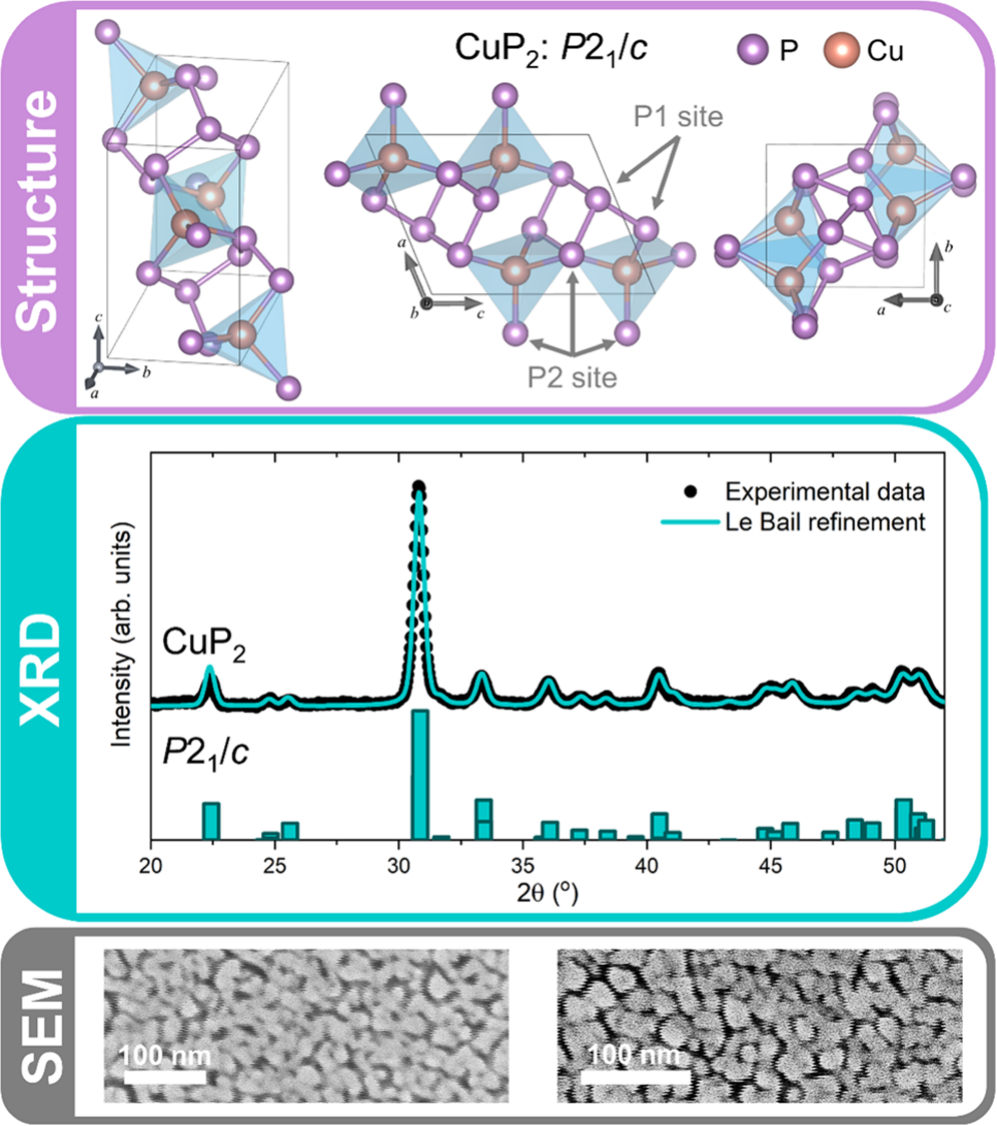
Structural and morphological characterization of CuP_2_ thin film. (Top) Crystal structure representation of monoclinic CuP_2_ unit cell along different crystal planes. (Middle) Le Bail refinement of the measured XRD pattern showing the presence of a single CuP_2_ phase (*P*2_1_/*c*) within the film. (Bottom) SEM top images of CuP_2_ thin film showing a porous polycrystalline morphology.

We start by providing evidence of the crystal structure and morphology of the sample investigated here. Figure 1 presents an overview of the structural and morphological characterization of the CuP_2_ thin film.

The top panel in Figure 1 shows the crystal structure of CuP_2_ by visualizing it from three different zone axes. The lattice has a monoclinic structure with *P*2_1_/*c* (*C*_2*h*_ (2/*m*)) space group. Cu is bonded in a tetrahedral geometry to four P atoms, with a spread of Cu–P bond distances ranging from 2.25–2.49 Å. In contrast, P atoms are distributed on two inequivalent sites, labeled P1 and P2 in Figure 1. In the P1 site, P is bonded to one Cu and three P atoms to form distorted PCuP_3_ tetrahedra, with a P–P bond length of 2.20 Å. In the P2 site, P is bonded to three equivalent Cu and two equivalent P atoms to form distorted PCu_3_P_2_ trigonal bipyramids, resulting in two different P–P bond lengths of 2.19 and 2.21 Å. Overall, the CuP_2_ structure is characterized by alternating layers of CuP_4_ tetrahedra and of homoelement-bonded P atoms along the [100] direction (*a*-axis).

The middle panel in Figure 1 presents the measured XRD pattern of the CuP_2_ thin film. Le Bail refinement confirmed the presence of a single CuP_2_ phase (*P*2_1_/*c*), without any additional crystalline phases. The determined lattice parameters are *a* = 5.80 ± 0.02 Å, *b* = 4.82 ± 0.02 Å, *c* = 7.53 ± 0.02 Å, α = 90°, β = 112.68 ± 0.02°, and γ = 90°. These are in good agreement with the results obtained from neutron diffraction experiments on CuP_2_ powders from ref (11). The reference XRD pattern of the CuP_2_ phase (*P*2_1_/*c*) is shown below the measured XRD data.

Finally, the surface morphology of the CuP_2_ thin films is shown in the bottom panel of Figure 1, where a porous polycrystalline matrix with grain size around 30 nm is observed.

### Raman Properties of CuP_2_: Calculations and Measurements

Group theory analysis predicts the following set of irreducible representations for the structure *P*2_1_/*c* (*C*_2*h*_ (2/*m*)) at the Γ point of the Brillouin zone^27−29^
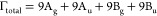
Raman and infrared active modes are
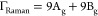

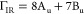
while 1A_u_ + 2B_u_ modes are acoustic modes. Note also that A and B refer to the nondegenerate symmetric and asymmetric modes with respect to the principal symmetry axis, respectively, while distinctions g and u correspond to symmetric or asymmetric vibrations with respect to the center of inversion. The Raman tensors for **P**2_1_/*c* space group^27−29^ are defined as follows:
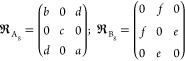
where *a*, *b*, *c*, *d*, *e*, and *f* are the Raman tensor elements.

**Figure 2:**
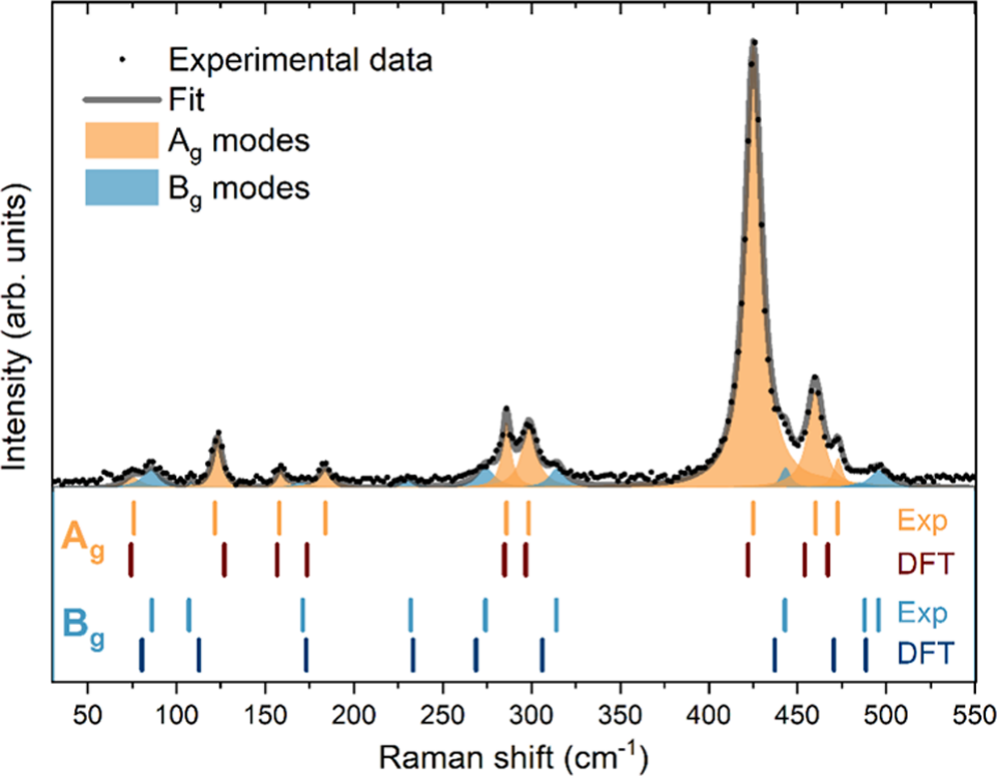
Lorentzian deconvolution of the experimental Raman spectrum measured on reference CuP_2_ thin film with 532 nm excitation. Vertical lines under the spectrum show a comparison between the Raman peak positions obtained experimentally by the deconvolution (labeled “exp”) and from the lattice dynamics calculations based on DFT (labeled “DFT”).

Figure 2 presents the Raman spectrum of CuP_2_ thin film measured with 532 nm excitation. Deconvolution of the Raman spectrum with a minimum number of Lorentzian components was performed, allowing identification of total 18 Raman peaks, as predicted by theory and shown in Figure 2. Each peak was modeled with a Lorentzian curve characterized with peak position, peak width, and intensity. As the fitting procedure includes a large number of variables, rigorous restrictions were imposed on the fitting parameters in order to avoid correlation among the parameters and obtain meaningful results. In this case, this included leaving the intensity and peak position as free parameters while the widths of peaks were restricted to certain conditions. As the peak widths are mostly dependent on the phonon lifetime, which is determined by the crystal quality of the material, it is expected that all fundamental one-phonon Raman modes have similar widths, regardless of the symmetry of the mode. This results in allowing only a narrow interval of change for the one-phonon peak widths during the whole deconvolution process. Possible two-phonon or multiphonon modes would be similarly modeled with double or multiple widths of the one-phonon modes. This leads to an unambiguous interpretation of the phonon nature of the peaks, rendering the identification procedure more accurate.

Table 1 lists the Raman frequencies of all peaks obtained from the deconvolution, comparison with DFT-calculated phonon modes, their symmetry assignment, and comparison with values provided in the literature.^6^ All peaks are identified as one-phonon modes based on the results from the deconvolution procedure. Overall, we observe an excellent agreement (within, on average, 2% difference) between the experimentally observed peaks and the theoretically predicted Raman frequencies. Minor disagreement in the Raman peak positions between the experimental and the theoretical results is expected, due to approximations applied during the calculations, such as the three-body and long-range interactions.

**Table 1:** Frequency (in cm^–1^) of Peaks from Lorentzian Fitting of CuP_2_ Raman Spectrum Measured with 532 nm Laser Excitation and Proposed Mode Symmetry Assignment Compared with Theoretical Predictions and References

this work			refs[^6^]
ν_exp_ (cm^–1^)	ν_theory_ (cm^–1^)	symmetry assignment	ν_exp_ (cm^–1^)
75	75	A_g_	
86	81	B_g_	
108	113	B_g_	
123	127	A_g_	121
158	157	A_g_	
170	173	B_g_	
184	173	A_g_	183
230	233	B_g_	
274	269	B_g_	
285	285	A_g_	286
298	297	A_g_	299
314	306	B_g_	
422	422	A_g_	425
443	437	B_g_	
460	454	A_g_	461
473	467	A_g_	472
485	471	B_g_	
496	489	B_g_	496

**Figure 3:**
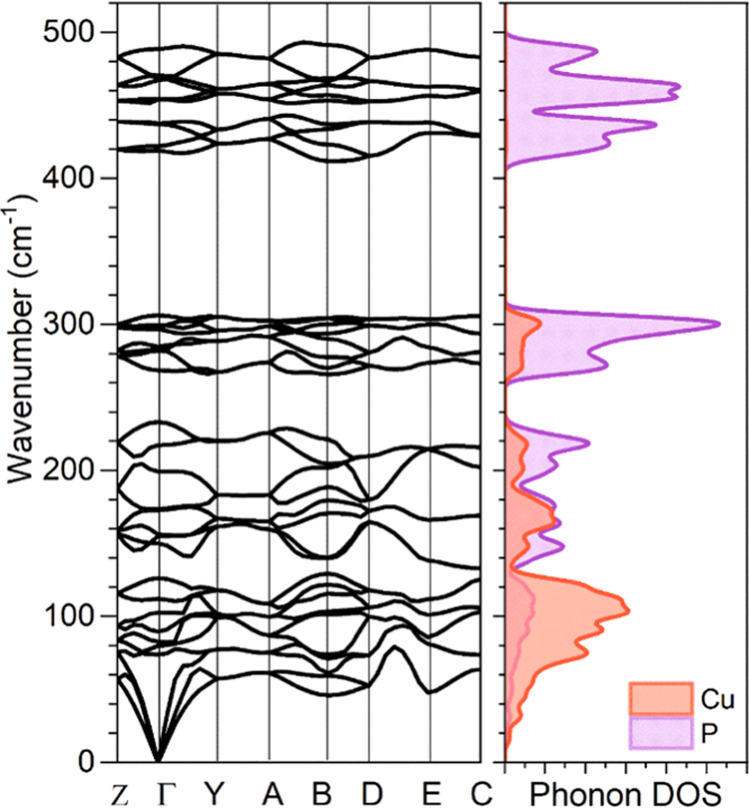
DFT-calculated phonon dispersion along high-symmetry directions of monoclinic CuP_2_ structure. On the right, the partial phonon density of states is presented: Cu atoms in red and P atoms in purple.

More detailed analysis of the CuP_2_ phonons can be obtained from the calculated phonon dispersion along high-symmetry directions of the Brillouin zone, which is presented in Figure 3, along with the elemental phonon density of states (PDOS). Several distinct regions can be identified in the phonon dispersion diagram: (i) the low-frequency region (<140 cm^–1^), which is mostly dominated by Cu-related vibrations, (ii) two intermediate regions (140–240 and 260–320 cm^–1^), which correspond to mixed contributions from Cu- and P-related vibrations, and (iii) the high-frequency region (400–500 cm^–1^), which is attributed mainly to P-related vibrations. Besides this, two-phonon gaps are observed, first in the 240–260 cm^–1^ frequency region and second in the 320–400 cm^–1^ region. The observation of the phonon gaps seems typical for XP_2_ compounds, as similar features were observed for ZnP_2_ and CdP_2_ materials.^30^ Additionally, we note that the position and shape across the Brillouin zone of those phonon band gaps seem virtually independent of the cations (Cu, Zn, or Cd), as it is usually found in the 300–400 cm^–1^ region. This feature could be further exploited for thermoelectric applications, for example.

**Figure 4:**
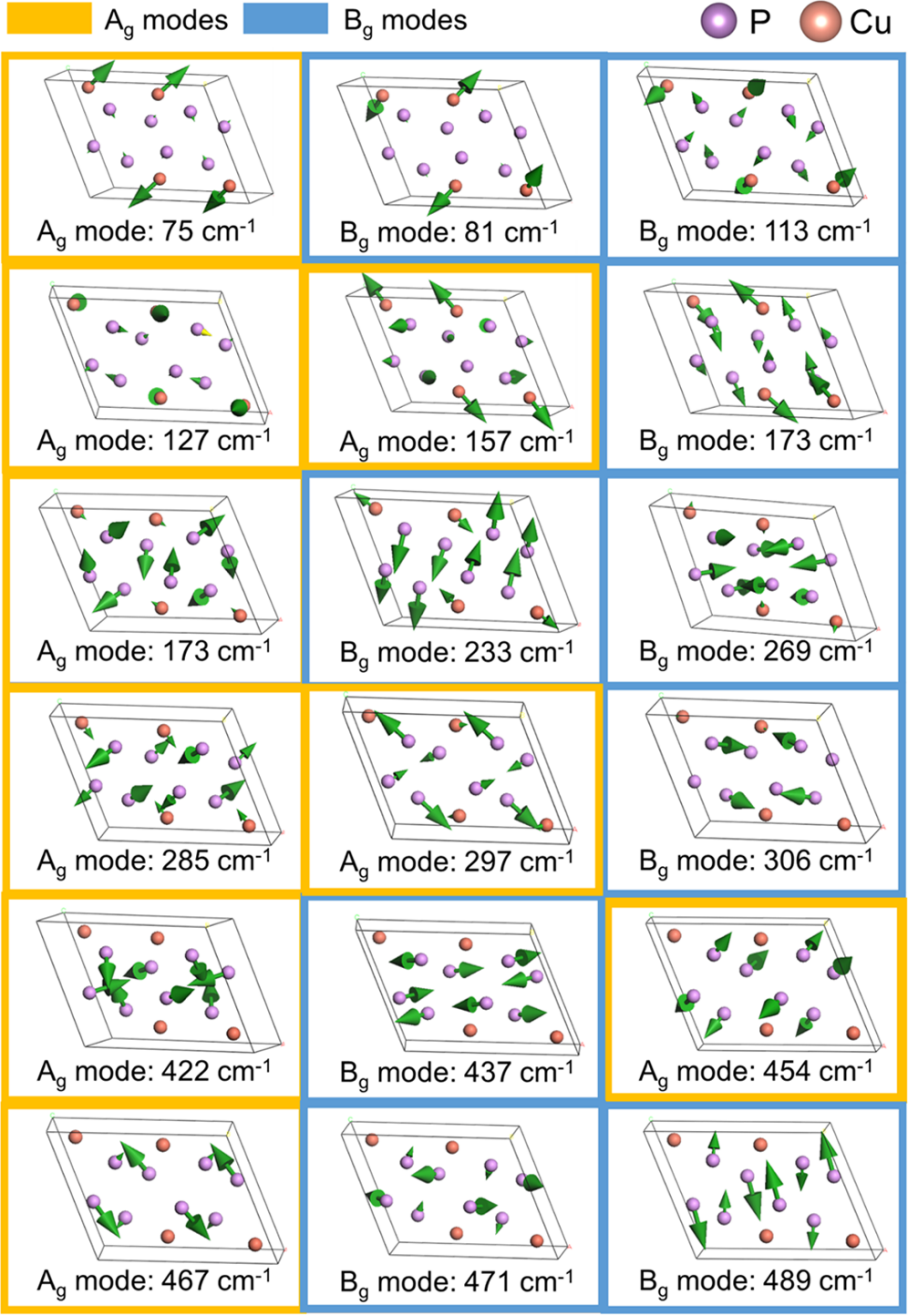
Calculated phonon displacements for Raman modes of CuP_2_. Mode symmetries and frequencies (in cm^–1^) are listed under each picture.

Atomic displacements of the Raman modes were calculated to provide the visualization of the corresponding atom motions. Figure 4 shows the vibrational patterns of all Raman active modes. As expected from the PDOS, the vibrational patterns are mostly dominated by either Cu (<120 cm^–1^) or P motions (>260 cm^–1^). The majority of B_g_ modes involve atomic motion parallel to the horizontal *ab* plane or vertical *ac* or *bc* planes. On the other hand, the A_g_ modes are characterized by a more complex behavior, for example, breathing-like vibrations of P atoms for the modes centered at 285, 422, and 454 cm^–1^ frequencies.

**Figure 5:**
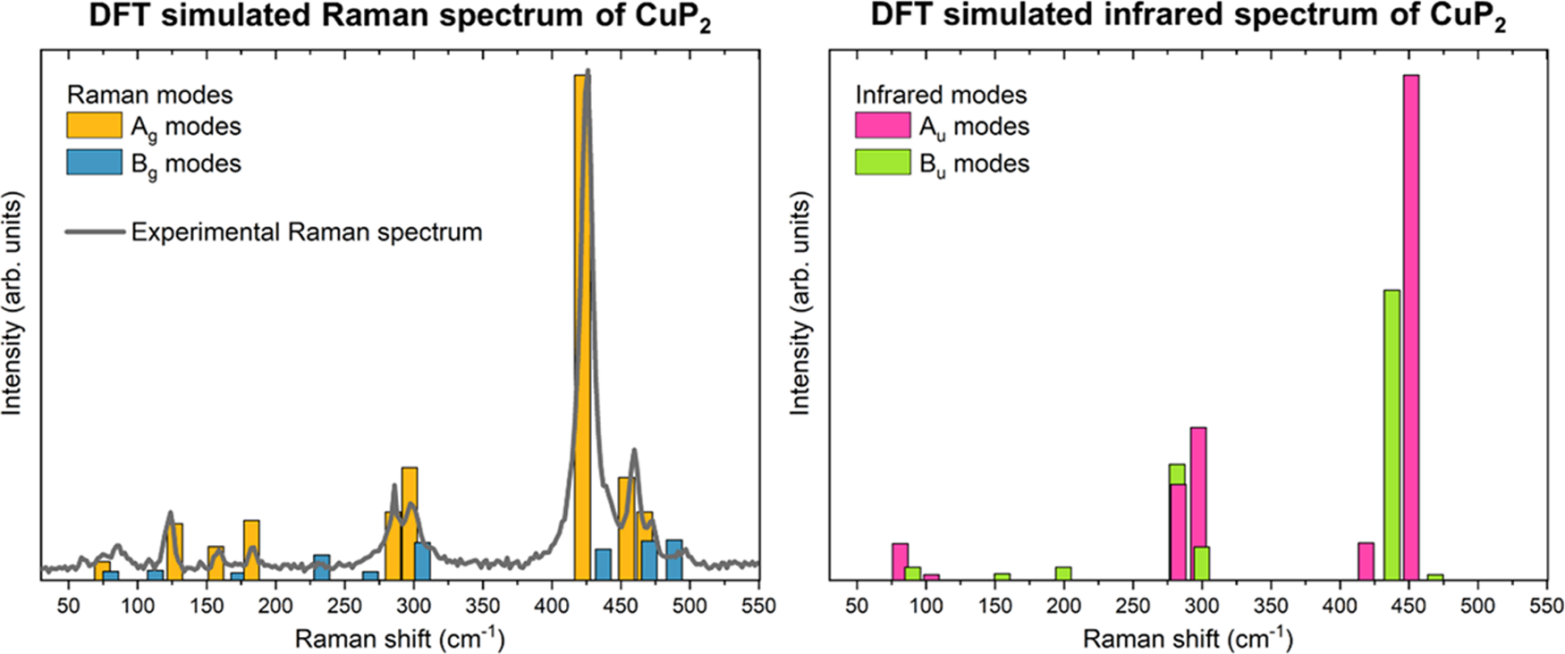
(Left) Comparison between the calculated intensity of the Raman modes (colored bars) and the experimental Raman spectrum (gray line). (Right) Calculated intensities of the IR active modes.

To validate the experimentally measured Raman spectrum as a reference for the CuP_2_ compound, we have calculated the Raman mode intensities using DFT and compared them to the experimental values. Nonresonant Raman intensities were calculated from the Raman tensor coefficient obtained from the first-order dielectric tensor for the equilibrium crystal configuration and for the crystal with atomic displacement according to the vibrational patterns of the individual phonon modes. These were then adjusted to our experimental conditions (λ_ext_ = 532 nm and *T* = 300 K) by using the coefficient *C*(ω_p_), which describes the dependence of the Raman mode intensity on the phonon frequency ω_p_ and the incident laser frequency ω_i_^13,31^
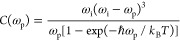
where ℏ = *h*/2π, with *h* being the Planck constant, *k*_B_ is the Boltzmann constant, and *T* is the temperature of the measurements. Figure 5(left) presents the comparison between the calculated intensities of the Raman modes and the experimental Raman spectrum. Overall, Figure 5(left) suggests a very good agreement between the theory and experiment, showing that the measured Raman spectrum can indeed be used as a reference for CuP_2_. Minor discrepancies are observed for the calculated intensities of the A_1g_ modes, which are slightly overestimated. There are several possible reasons for these kinds of discrepancies. The first reason is related to the way Raman intensities are calculated in DFT, where certain approximations are necessary for making feasible calculations. These include approximations in the many-body interactions, which can become especially important for structures with large number of atoms, such as CuP_2_, or the overestimation of the polarizability in semilocal DFT.^32,33^ Other possible sources of mismatch in the simulations include the use of an ideal periodic crystal with no treatment of defects or disorder. Defects especially can affect the intensities of Raman modes.^19−21^ Considering that the measured CuP_2_ is in polycrystalline form, it is possible that the increased concentration of structural defects is affecting the intensities of certain modes in the Raman spectra, thus contributing to the discrepancy between the experimental and theoretical results.

### Infrared (IR) Properties of CuP_2_

Finally, Figure 5(right) presents the calculated intensities of the IR active modes, while Table 2 lists the positions of these modes, along with their symmetry. Here, it is interesting to note that the rattling mode corresponding to Cu atomic dimers vibrations with frequency around 11 meV (89 cm^–1^) observed by Qi et al.^11^ is in very good agreement with the calculated IR active B_u_ mode at 90 cm^–1^.

**Table 2:** Frequency (in cm^–1^) and Corresponding Symmetry of the IR Active Modes Obtained from the DFT Calculations

ν_theory_ (cm^–1^)	symmetry assignment
81	A_u_
90	B_u_
104	A_u_
150	A_u_
155	B_u_
199	B_u_
218	A_u_
282	B_u_
283	A_u_
287	A_u_
300	B_u_
419	A_u_
438	B_u_
452	A_u_
469	B_u_

## Conclusions

Vibrational properties of stoichiometric CuP_2_ were reported from Raman measurements and first-principles calculations. Particular focus was put on the detailed deconvolution of the Raman spectrum with Lorentzian curves, which has allowed identification of all theoretically predicted Raman active modes (9A_g_ and 9B_g_), including their positions and symmetry assignment. Furthermore, calculations of the phonon density of states (PDOS), as well as the phonon dispersions, provide a microscopic understanding of the experimentally observed phonon lines, in addition to the assignment to the specific lattice eigenmodes. We further provide the theoretically predicted positions of the infrared (IR) active modes, along with the simulated IR spectrum. Overall good agreement is established between the experimental and theoretically calculated Raman spectra of CuP_2_, providing a reference platform for future investigations on this material. We suggest using the most intense Raman peaks located around 123, 184, 285, 298, 422, 460, 473, and 496 cm^–1^ as reference for identification of the Cu_2_P phase.
